# Photothermal therapy with immune-adjuvant nanoparticles together with checkpoint blockade for effective cancer immunotherapy

**DOI:** 10.1038/ncomms13193

**Published:** 2016-10-21

**Authors:** Qian Chen, Ligeng Xu, Chao Liang, Chao Wang, Rui Peng, Zhuang Liu

**Affiliations:** 1 Institute of Functional Nano and Soft Materials (FUNSOM), Jiangsu Key Laboratory for Carbon-Based Functional Materials and Devices, Soochow University, Suzhou, Jiangsu 215123, China

## Abstract

A therapeutic strategy that can eliminate primary tumours, inhibit metastases, and prevent tumour relapses is developed herein by combining adjuvant nanoparticle-based photothermal therapy with checkpoint-blockade immunotherapy. Indocyanine green (ICG), a photothermal agent, and imiquimod (R837), a Toll-like-receptor-7 agonist, are co-encapsulated by poly(lactic-co-glycolic) acid (PLGA). The formed PLGA-ICG-R837 nanoparticles composed purely by three clinically approved components can be used for near-infrared laser-triggered photothermal ablation of primary tumours, generating tumour-associated antigens, which in the presence of R837-containing nanoparticles as the adjuvant can show vaccine-like functions. In combination with the checkpoint-blockade using anti-cytotoxic T-lymphocyte antigen-4 (CTLA4), the generated immunological responses will be able to attack remaining tumour cells in mice, useful in metastasis inhibition, and may potentially be applicable for various types of tumour models. Furthermore, such strategy offers a strong immunological memory effect, which can provide protection against tumour rechallenging post elimination of their initial tumours.

## Abstract (web summary)

Photothermal therapy can induce an anti-tumour immune response by producing tumour-associated antigens. Here, the authors design a nanoparticle that simultaneously acts as a photothermal agent and an immune-adjuvant and demonstrate the anti-tumour efficacy in combination with anti-CTLA4 therapy in preclinical murine cancer models.

## Graphical abstract

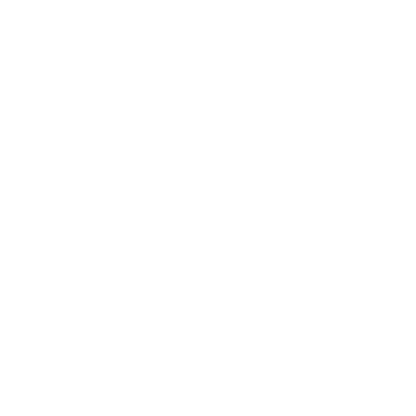

Developing effective therapeutic strategies with high specificities and low toxicities to eradicate tumours, particularly post their metastases, and further prevent their recurrence, is the ultimate goal in the battle against cancer. Currently used gold-standard cancer treatment approaches, surgery chemotherapy and radiotherapy, all fail to achieve this goal. In recent years, along with the growing knowledge on cancers and their interactions with immune systems, cancer immunotherapy by training or stimulating the inherent immunological systems of the body to attack tumour cells, has been progressing rapidly and shown tremendous promises as a next generation of cancer treatment strategy^1^,^2^. Several different types of cancer immunotherapies including cytokine therapy^3^,^4^, checkpoint-blockade therapy^5^,^6^, adoptive T-cell transfer especially the emerging chimeric antigen receptor T (CAR-T) cell therapy^7^,^8^,^9^,^10^,^11^, as well as cancer vaccines^12^,^13^,^14^, have demonstrated some exciting clinical responses. However, until now, most of those immune-therapeutic strategies still have limitations such as extremely high costs^15^, large individual variations in therapeutic responses, as well as certain immunotoxicity like the cytokine release syndrome^14^,^16^,^17^.

Among above-mentioned cancer immunotherapy strategies, cancer vaccines may own a number of unique advantages^18^,^19^. It has been demonstrated that cancer vaccines loaded with tumour-associated antigens are able to induce antigen-specific immunities against tumours, rather than non-specific immunological responses triggered by other methods such as the checkpoint-blockade therapy^20^. On the other hand, cancer vaccines may offer a long-term immune-memory effect that could be helpful to prevent cancer reccurrence^20^,^21^,^22^. In general, cancer vaccines involve tumour-specific antigens-based vaccines and whole cancer cell vaccines^23^,^24^. Although tumour-associated antigens such as specific proteins or peptides with the help of adjuvant agents may induce robust anti-tumour immune responses, the large heterogeneity of patients lead to their limited clinical applications^25^. Different from the former, whole cancer cell vaccines (for example, using lysates of dissected tumour tissues) can induce immunities against all released potential tumour antigens, and in principle should be applicable to various types of solid tumours^18^. However, the complicated manufacture process, the uncertainties in characteristics and dosages for whole cancer cell vaccines, as well as their limited efficacies resulted in disappointing clinical results so far^26^. Therefore, a cancer immunotherapy strategy that is easy to operate and has high specificity and efficacy is urgently needed.

Photothermal therapy (PTT) has been developed as a new cancer treatment strategy that employs the heat generated from the absorbed optical energy by light-absorbing agents accumulated in the tumour to ablate tumour cells^27^,^28^. Recently, we and others^29^,^30^,^31^,^32^,^33^ discovered that photothermal therapy with inorganic nano-agents (for example, carbon nanotubes, graphene oxide or CuS nanoparticles) could generate anti-tumour immunological effects by producing tumour-associate agents from ablated tumour cell residues. Such an effect has also been observed in a preliminary clinical trial study^32^. Inspired by such interesting findings, in this work we discover that the tumour-associated antigens generated *in situ* after photothermal tumour ablation in the presence of immune-adjuvant nanoparticles could show vaccine-like functions, which in combination with checkpoint blockade show strong anti-tumour immune responses for effective cancer immunotherapy (Fig. 1a).

In our formulation, those nanoparticles are composed by three US FDA (Food and Drug Administration) -approved agents, poly(lactic-co-glycolic) acid (PLGA) as the encapsulating polymer, indocyanine green (ICG) as the near-infrared (NIR) dye to enable photothermal therapy, and imiquimod (R837) which is a potent TLR7 agonist useful in activating immune responses^34^,^35^. Upon NIR-induced photothermal ablation of primary tumours injected with PLGA-ICG-R837, the released tumour-associated antigens in combination with R837-loaded nanoparticle adjuvant would show vaccine-like functions, generating strong immunological responses which with the help of anti-CTLA4 checkpoint-blockade therapy to inhibit the activities of immune-suppressive regulatory T cells (Tregs) could attack distant tumour cells remaining in the mouse body. This strategy appears to be particularly effective in inhibiting tumour metastasis post spreading of tumour cells in the mouse body. PLGA-ICG-R837-based photothermal treatment combined with anti-CTLA4 therapy could protect treated mice against tumour cells rechallenging 40 days post ablation of primary tumours, demonstrating the strong immune-memory effect to protect mice from cancer relapse. Such a strategy could also work by systemic injection of reformulated nanoparticles to realize effective cancer treatment. Therefore, the use of immune-adjuvant nanoparticles for photothermal tumour ablation offers vaccine-like functions *in situ,* which in combination with the clinically adapted checkpoint-blockade method shows efficacy in cancer immunotherapy.

## Results

### Nanoparticle formulation and immune-stimulation abilities

**Figure 1:**
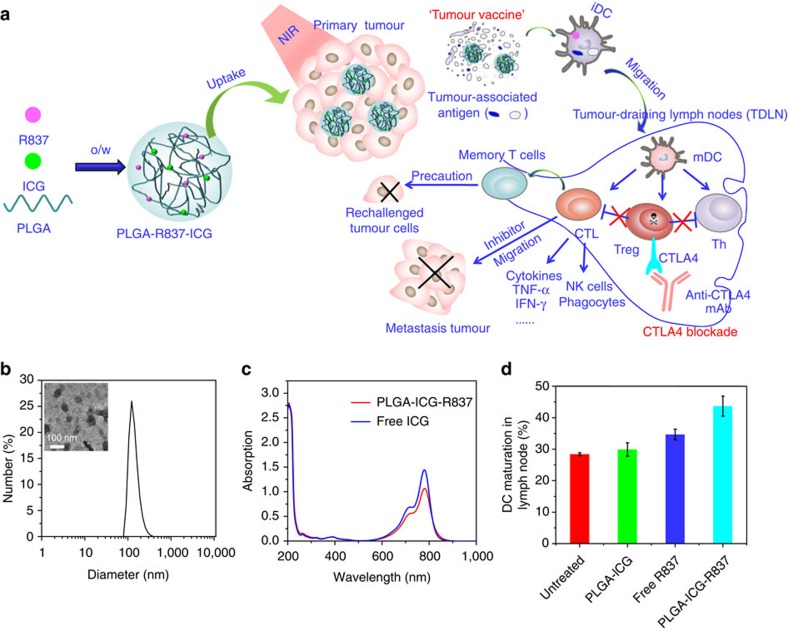
Formulation of nanoparticles and their immune-stimulation abilities. (**a**) The mechanism of anti-tumour immune responses induced by PLGA-ICG-R837-based PTT in combination with checkpoint-blockade. (**b**) Hydrodynamic diameters of PLGA-ICG-R837 nanoparticles measured by DLS. Inset: (**a**) TEM image of PLGA-ICG-R837. (**c**) UV–vis–NIR spectra of PLGA-ICG-R837 and free ICG, indicating the successful loading of ICG into PLGA. (**d**) *In vivo* DC maturation (CD80+CD86+) with lymph nodes of BALB/c mice s.c. injected with PLGA-ICG, free R837, or PLGA-ICG-R837 (three mice per group). Data are presented as the mean±s.e.m. Error bars are based on triplicated experiments. DLS, dynamic light scattering; TEM, transmission electron microscopy.

PLGA, a biodegradable synthetic polymer approved for clinical use by US FDA, was used to encapsulate two types of small molecules, a NIR dye ICG and a TLR7 ligand R837, by oil-in-water (o/w) emulsion method. The obtained PLGA-ICG-R837 nanoparticles showed well-defined spherical shape and homogenous sizes as revealed in the transmission electron microscope image (Fig. 1b, inset). The average hydrodynamic size of PLGA-ICG-R837 was ∼100 nm as measured by the dynamic light scattering (Fig. 1b). The ultraviolet–visible–NIR absorption spectrum of PLGA-ICG-R837 showed the characteristic absorption peak of ICG, indicating the successful encapsulation of ICG in the PLGA core (Fig. 1c). The loading of R837 was confirmed by high-performance liquid chromatography (Supplementary Fig. 1).

As one of the most important classes of antigen-presenting cells, DCs play crucial roles in initiating and regulating the innate and adaptive immunities^36^. Upon exposure to antigens, immature DCs will engulf and then process them into peptides during their migration to nearby lymph nodes. Thereafter, those immature DCs would transform into mature DCs and present the major histocompatibility complex-peptide to T-cell receptor when arriving at lymph nodes^37^. Therefore, we firstly investigated the immunological effects of PLGA-ICG-R837 nanoparticles towards bone marrow-derived DCs separated from BALB/c mice, by using flow cytometry to analyse the upregulation of co-stimulatory molecules CD80, CD86, which are well-known markers for DC maturation. It was found that PLGA-ICG-R837 nanoparticles could greatly promote *in vitro* DC maturation similar to free R837 at the same dose, while PLGA-ICG showed no obvious immune-stimulation effect to DCs (Supplementary Fig. 2).

To further investigate if PLGA-ICG-R837 could accelerate DC maturation *in vivo*, BALB/c mice were subcutaneously (s.c.) injected with PLGA-ICG, free R837, PLGA-ICG-R837 (50 mg kg^−1^ PLGA, 0.7 mg kg^−1^ R837, 1.1 mg kg^−1^ ICG). Three days post injection, mice were killed and the inguinal lymph nodes were collected for assessment by flow cytometry after co-staining with CD11c (the DC marker), CD80 and CD86. The percentage of matured DCs (CD11c+CD80+CD86+) significantly increased from ∼28 to ∼45% after treatment with PLGA-ICG-R837, while the DC maturation percentages from mice treated with PLGA-ICG or free R837 (with the same dose) only increased to ∼30 or ∼35%, respectively. Therefore, PLGA-ICG-R837 nanoparticles showed even stronger *in vivo* immune-stimulation effect compared with the same dose of free R837, although the two induced similar levels of *in vitro* DC maturation (Fig. 1d and Supplementary Fig. 3).

DCs upon maturation would secrete various types of cytokines to regulate other types of immune cells^38^. Thus, in the following experiment, various cytokines including interleukin 6 (IL-6) (an important marker in the activation of humoral immunity), tumour necrosis factor α (TNF-α) (an important marker in the activation of cellular immunity), and interleukin 12 (IL-12p70) (an important marker of innate immunity)^39^,^40^,^41^,^42^ in the mouse sera after different treatment were analysed by ELISA. It was found that mice treated with PLGA-ICG-R837 showed high serum levels of IL-12p70, IL-6 and TNF-α, which appeared to be higher than those in sera of mice treated with the same dose of free R837 (Supplementary Fig. 4). Such observed stronger *in vivo* immune-stimulation effect of PLGA-ICG-R837 than free R837 may be attributed to the sustained release of R837 from nanoparticles.

### Photothermal tumour ablation for immune system activation

On the basis of the aforementioned experiment results, PLGA-ICG-R837 nanoparticles designed in our system is an effective immune-stimulator. It has been reported that many other ablative tumour treatments such as hyperthermia, photodynamic therapy and cryoablation will induce strong tumour-specific immune responses^43^,^44^,^45^,^46^. Therefore, we wonder if photothermal therapy with our PLGA-ICG-R837 could trigger further enhanced immunological responses. Firstly, *in vitro* experiments verified that the residues of 4T1 breast tumour cells after NIR-induced photothermal ablation with PLGA-ICG-R837 could dramatically enhance the DC maturation, to a level much higher than that achieved by simply adding PLGA-ICG-R837 nanoparticles, or cell residues ablated by PLGA-ICG in the absence of R837 (Supplementary Fig. 2). Such results suggest that R837-containing nanoparticles could potentially act as an adjuvant to promote immunological responses of tumour-associate antigens in cell residues.

**Figure 2:**
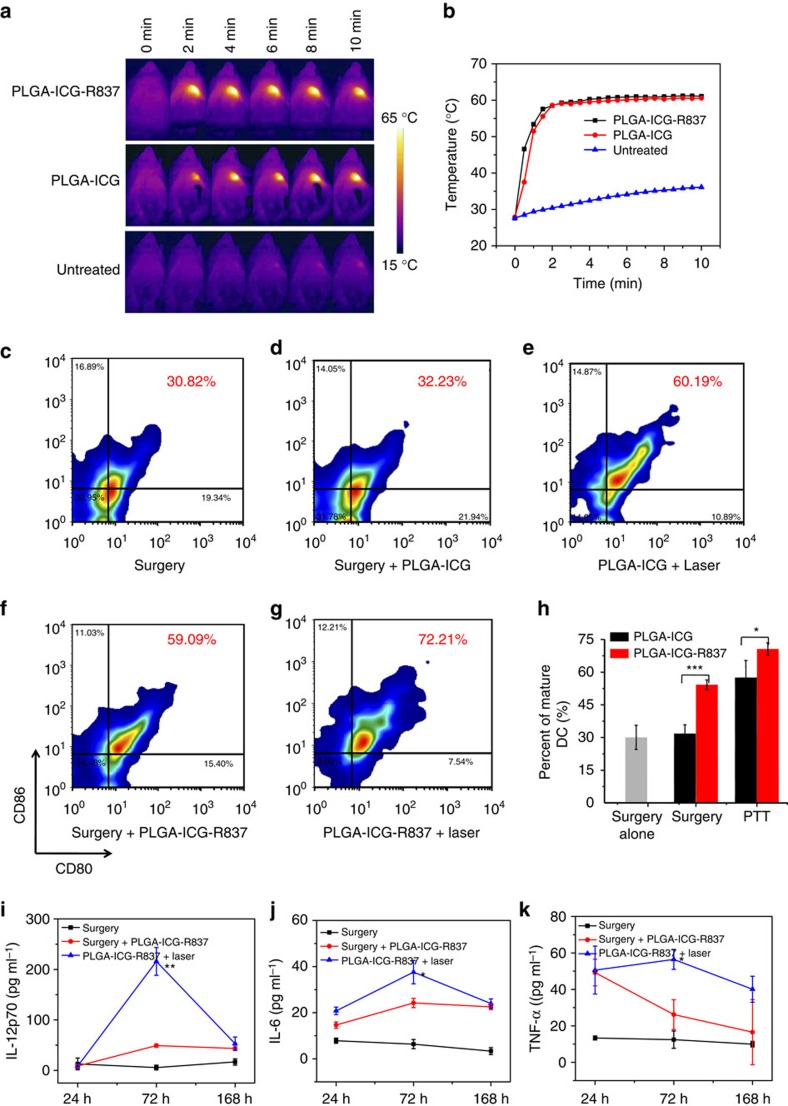
Immune responses after PLGA-ICG-R837-based PTT. (**a**) IR thermal images of 4T1-tumour-bearing mice injected with PLGA-ICG-R837, PLGA-ICG or PBS under the 808 nm laser (0.5 W cm^−2^) irradiation. (**b**) The tumour temperature changes based on IR thermal imaging date in **a**. (**c**–**h**) DC maturation induced by PLGA-ICG-R837-based PTT on mice-bearing 4T1 tumours (gated on CD11c+ DC cells). Cells in the tumour-draining lymph nodes were collected 72 h after various treatments for assessment by flow cytometry after staining with CD11c, CD80 and CD86. (**i**–**k**) Cytokine levels in sera from mice isolated at 24, 72 and 168 h post different treatments (surgery, surgery and s.c. injection of PLGA-ICG-R837, i.t. injection of PLGA-ICG-R837 and PTT). Three mice were measured in each group in (**a**–**k**). Data are presented as the mean±s.e.m. *P* values were calculated by Tukey's *post-hoc* test (***P<0.001, **P<0.01 or *P<0.05). For (**i**–**k**), *P* values were determined between the second group (Surgery+PLGA-ICG-R837) and the third group (PLGA-ICG-R837+laser).

In our further *in vivo* experiment, BALB/c mice-bearing subcutaneous 4T1 tumours were intratumourally (i.t.) injected with PLGA-ICG or PLGA-ICG-R837 and then irradiated by an 808 nm laser at the power density of 0.5 W cm^−2^ for 10 min. As monitored by an infrared thermal camera (Fotric 225), the tumour temperature of mice injected with PLGA-ICG or PLGA-ICG-R837 under laser irradiation quickly rose to ∼60 °C, which was high enough to effectively ablate tumours (Fig. 2a,b). To analyse the status of DCs in treated tumours after PTT, tumour cells were collected and co-stained with CD11c/propidium iodide for assessment by flow cytometry 4 h post-PTT treatment with PLGA-ICG-R837. After photothermal ablation of the tumour with PLGA-ICG-R837 nanoparticles, more DCs would be recruited into the initial tumour site, although DCs pre-existing in the tumour before treatment might have been killed alongside the tumour (Supplementary Fig. 5). Three days after photothermal therapy, mice were killed with their tumour-draining lymph nodes collected for assessment using flow cytometry after co-staining with various markers. Interestingly, photothermal tumour ablation with adjuvant nanoparticles (PLGA-ICG-R837+Laser) induced an high level of DC maturation (∼72%), which appeared to be much higher than that observed for adjuvant nanoparticles alone (PLGA-ICG-R837 without laser) or PTT with PLGA-ICG in the absence of immune-adjuvant (Fig. 2c–h). Therefore, after the tumour is destroyed post PTT, DCs may be recruited to the ablated tumour site as antigen-presenting cells to trigger immune responses. Meanwhile, tumour-associated antigens in tumour debris post PTT may be transported into nearby lymph nodes and then processed by DCs to simulate DC maturation, particularly with the help of adjuvant nanoparticles.

Cytokines secretion is also important in the process of immune responses. In a parallel experiment, sera of mice bearing either 4T1 tumours or CT26 tumours after different treatments were collected at 24, 72 and 168 h to analyse the changes of various cytokines including IL-6, TNF-α and IL-12p70. Similarly, although PLGA-ICG-R837 injection alone or PTT with PLGA-ICG was able to increase the secretion of pro-inflammatory cytokines, their secretions induced by PLGA-ICG-R837-based PTT were obviously higher and lasted longer, favourable for triggering anti-tumour immune response (Fig. 2i–k and Supplementary Fig. 6). The non significant increase of IL-4 secretion, an important indicator for humoral immunity, in those treated mice (Supplementary Fig. 7), indicates that humoral immunity may play a less important role in this system. Considering the risk of high cytokine levels to induce possible harmful effects to normal organs, serum biochemistry assay and complete blood panel test were conducted for 4T1-tumour-bearing mice at 1, 7 and 14 days after PLGA-ICG-R837-based photothermal therapy. All measured parameters fell within normal ranges, indicating that such elevated cytokine levels post PTT with PLGA-ICG-R837 should be well tolerable by those mice (Supplementary Table 1). These results suggest that PLGA ICG R837-based photothermal therapy is able to induce immunological stimulation effects *in vivo*. We thus hypothesize that the *in vivo* adjuvant activities of those R837-containing nanoparticles in combination with tumour-associate antigens released after tumour ablation therapy could act together as a safe ‘tumour vaccine' potentially useful for cancer immunotherapy.

### PTT plus CTLA4 blockade to inhibit growth of distant tumours

The majority of cancer deaths are caused by metastases, which if occurred can hardly be effectively treated by conventional therapies such as surgery, chemotherapy and radiotherapy^47^,^48^. Therefore, we wondered if photothermal immunotherapy with our PLGA-ICG-R837 could provide any opportunity in treating metastatic cancer. Cytotoxic T lymphocyte-associate antigen-4 (CTLA-4) is a critical negative regulator of immune responses^49^,^50^, and its blockade by antibodies (for example, anti-CTLA-4) to inhibit the activities of immune-suppressive Tregs has been approved by FDA as a cancer immunotherapy approach currently used in the clinic^51^,^52^,^53^,^54^. Therefore, in our animal experiments, CTLA-4 blockade therapy was introduced, aiming at enhancing the anti-cancer therapeutic efficacy of ‘tumour vaccines' that are *in situ* generated after PLGA-ICG-R837-based photothermal ablation of primary tumours.

**Figure 3:**
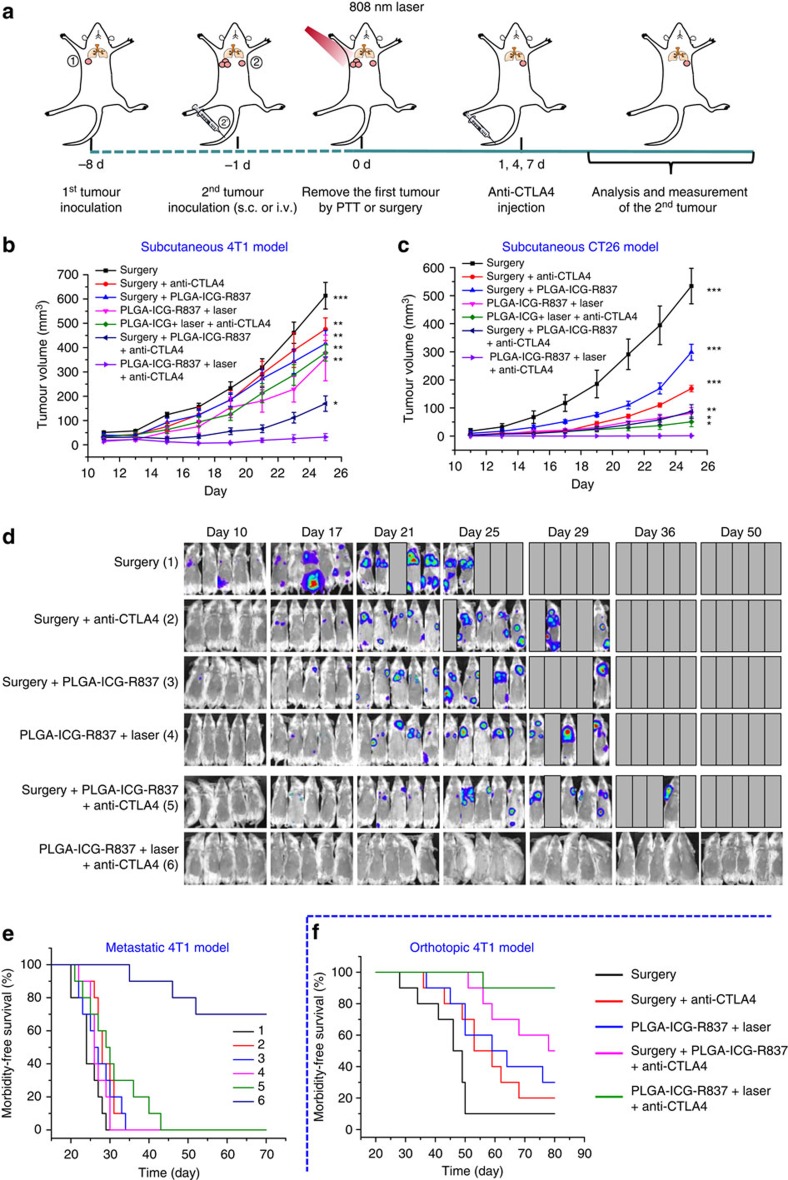
Anti-tumour effect of PLGA-ICG-R837-based PTT plus anti-CTLA-4 therapy. (**a**) Schematic illustration of PLGA-ICG-R837-based PTT and anti-CTLA-4 combination therapy to inhibit tumour growth at distant sites. (**b**,**c**) Tumour growth curves of different groups of mice (six mice per group) with s.c. inoculation of secondary 4T1 (**b**) or CT26 (**c**) tumours after various treatments to eliminate their primary tumours. (**d**) *In vivo* bioluminescence images to track the spreading and growth of i.v. injected fLuc-4T1 cancer cells in different groups of mice after the cancer cells after various treatments to eliminate their primary tumours. (**e**) Morbidity-free survival of different groups of mice with metastatic 4T1 tumours in **d** after various treatments indicated to eliminate their primary tumours (10 mice per group). (**f**) Morbidity-free survival of different groups of mice-bearing orthotopic 4T1 tumours with spontaneous metastases after various treatments indicated to eliminate their primary breast tumours (10 mice per group). PLGA-ICG-R837-based photothermal ablation of the first primary tumours in combination with anti-CTLA4 treatment would be able to induce strong anti-tumour immunological effects to inhibit the growth of tumour cells spread into other organs. *P* values in **b** and **c** were calculated by Tukey's *post-hoc* test (***P<0.001, **P<0.01 or *P<0.05) by comparing other groups with the last group (PLGA-ICG-R837+laser+anti-CLTA-4). Data are presented as the mean±s.e.m.

The design of our animal experiment is shown in Fig. 3a. Tumour cells including breast cancer (4T1) and colorectal cancer (CT26) were inoculated on the left flank of each mouse. A week later, a second tumour was inoculated on the right flank of the same mouse as an artificial mimic of metastasis. In the following day, the first tumours were eliminated by PLGA-ICG-R837-based photothermal therapy or surgery. Afterwards, mice were intravenously (i.v.) injected with anti-CTLA4 (clone 9H10) at doses of 10 μg per mouse three times on day 1, 4 and 7 (ref. 50). The growth of secondary tumours in different groups was measured by a caliper every other day (Fig. 3b,c). For mice with their primary tumours removed by surgery, the secondary tumours in both tumour model showed rather rapid growth, whose speed could only be slightly delayed if the mice were either s.c. injected with PLGA-ICG-R837, or i.v. injected with anti-CTLA4. The combination treatment by both PLGA-ICG-R837 and anti-CTLA4, however, could significantly slow down the growth of secondary tumours (especially in the first 20 days) on mice with their primary tumours dissected by surgery, indicating that such non-specific combined immunotherapy could be effective in cancer treatment. On the other hand, for mice with their primary tumours ablated by PLGA-ICG-R837-based PTT, the growth of their secondary tumours was also partly delayed. We found that for mice with their primary tumours eliminated by PLGA-ICG-R837-based PTT in combination with CTLA-4 blockade, their secondary tumours showed almost completely inhibited growth for the 4T1 model, and disappeared for the C26T model, achieving efficacies much better than that obtained in the surgery groups receiving the combined PLGA-ICG-R837 plus anti-CTLA4 treatment. As another control, the immunological response induced by PLGA-ICG-based PTT of primary tumours (no R837) together with CTLA-4 blockade therapy could only inhibit the growth of secondary tumours in the early days, demonstrating the important role of the immune-adjuvant R837 in those nanoparticles to trigger strong immune responses.

In addition to the above subcutaneous tumour models, we further tested the efficacy of our method in the treatment of a more aggressive whole-body spreading tumour model. In this experiment, while the treatment plan was not changed, the second wave of tumour cells was induced by i.v. injection of 4T1 cells expressing firefly luciferase(fLuc-4T1) into mice before their primary tumours are eliminated either by surgery or PLGA-ICG-R837-based PTT (Fig. 3a). In the following days, mice were i.v. injected with anti-CTLA-4 antibody as aforementioned (dose=20 μg per mouse for each time). Considering the much higher aggressiveness of the lung metastasis tumour model, the dose of anti-CTLA-4 antibody used here was doubled. *In vivo* bioluminescence imaging was conducted to track the spreading and growth of fLuc-4T1 cancer cells in different groups of mice. From bioluminescence imaging, it was found that mice with their primary tumours removed by surgery showed obvious cancer metastasis 10 days after i.v. injection with fLuc-4T1 cells. For the other groups of mice with surgical removal of primary tumours and treated with PLGA-ICG-R837 alone, anti-CTLA4 alone or even the combination of PLGA-ICG-R837 and anti-CTLA4, significant bioluminescence signals, indication of tumour metastases, also showed up, although at later stages. In contrast, while mice treated with PLGA-ICG-R837-based PTT to ablate their primary tumours showed delayed metastases, the group with PLGA-ICG-R837-based PTT together with CTLA-4 blockade therapy showed nearly no metastasis (Fig. 3d). Photographs of India-ink stained whole lungs harvested at different days also confirmed that different from all control groups in which many metastatic tumour nodules were found in the mouse lung, no noticeable sign of lung metastasis was noted in the last group with PLGA-ICG-R837-based PTT combined with anti-CTLA-4 therapy (Supplementary Fig. 8).

To further evaluate the therapeutic outcomes of combined PLGA-ICG-R837-based PTT and anti-CTLA-4 therapy, mice after various treatments were closely monitored. We found 7 seven out of 10 mice receiving i.v. injection of 4T1 cells could survive for 70 days after PLGA-ICG-R837-based PTT plus anti-CTLA4 therapy (Fig. 3e), in marked contrast to mice in the other five control groups which all died within 25–40 days. The survived seven mice in this last group behaved normally and were killed at day 70 for careful necropsy, which uncovered no noticeable metastatic tumours in these seven mice.

In addition to the metastatic tumour model induced artificially, an orthotopic murine breast cancer model with spontaneous metastasis was created and used to evaluate the therapeutic efficacy of our treatment strategy. As shown in Supplementary Fig. 9a, fLuc-4T1 tumour cells were inoculated into the breast pad of each mouse. Two weeks later, when spontaneous metastases of tumour cells should have occurred, the primary tumour on each mouse was eliminated by PLGA-ICG-R837-based photothermal therapy or surgery. Afterwards, anti-CTLA4 with the same dose was i.v. injected (dose=20 μg per mouse for each time). Then, the *in vivo* bioluminescence imaging was conducted to track the metastases of fLuc-4T1 tumour cells after different treatments. It was also found that mice after PTT based on PLGA-ICG-R837 together with CTLA-4 blockade therapy showed effective inhibition of cancer metastasis, in marked contrast to other control groups in which detectable metastases were observed, sooner or later after treatments (Supplementary Fig. 9b). Those mice after various treatments were closely monitored. In comparison with different control groups, in which the majority or large proportions of mice died from spontaneous metastases within 80 days, combining PLGA-ICG-R837-based PTT with anti-CTLA-4 therapy resulted in 90% of survival rate in our observation period of 80 days (Fig. 3f). Therefore, it is obviously that immunological responses triggered after PLGA-ICG-R837-based photothermal ablation of primary tumours in combination with anti-CTLA-4 therapy can effectively inhibit cancer metastasis and prolong the survival of mice with spreading tumour cells.

Besides CTLA-4, programmed death 1 (PD-1), as another important T-cell inhibitory receptor, and PD-L1, one of its ligands, play important roles in helping cancer cells to evade the immune attack. Blockade of PD-L1, sometimes in combination with CTLA-4 blockade, to enhance anti-tumour activity has been approved for cancer immunotherapy in the clinic^50^,^55^. In our experiments, mice post spreading of 4T1 tumour cells (i.v. injection) with surgical removal of their primary tumours and treated with the combination of CTLA-4 and PD-L1 co-blockade, or even together with the treatment of PLGA-ICG-R837 but no laser irradiation, showed obvious cancer metastases as indicated by bioluminescence imaging (Supplementary Fig. 10). Therefore, our current strategy by combining nano-adjuvant-based PTT with anti-CTLA-4 appears to be more effective than the clinically adopted PD-L1+CTLA-4 co-blockade therapy. However, when we combined anti-PD-L1 and anti-CTLA-4 to treat mice with tumours after NIR-induced photothermal ablation with PLGA-ICG-R837, more than a half of mice died while no mice died after PLGA-ICG-R837-based PTT combined with single checkpoint blockade. Considering the critical roles of PD-L1 and CTLA-4 in the immune homoeostasis, the simultaneous blockade of these two checkpoints may lead to the immune-related adverse events or cytokine release syndrome^56^, which after PLGA-ICG-R837-based PTT may be beyond the limit that mice could tolerant.

### The mechanism study

**Figure 4:**
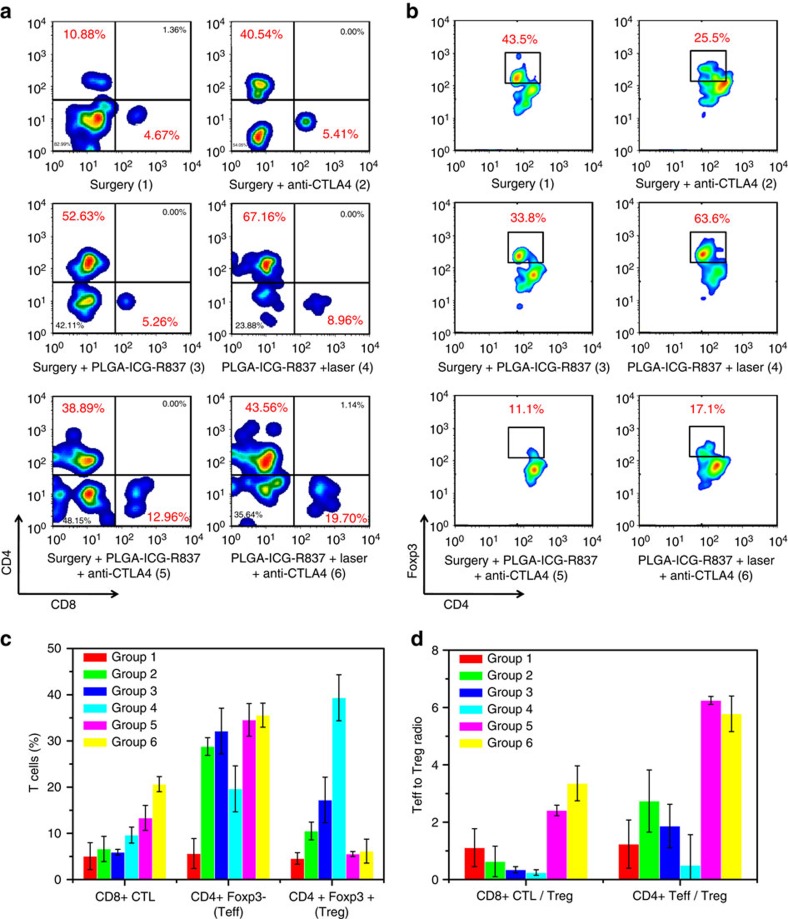
The mechanism study. (**a**) Representative flow cytometry plots showing different groups of T cells in secondary tumours. Tumour cell suspensions were analysed by flow cytometry for T-cell infiltration (gated on CD3+ T cells). (**b**) Representative flow cytometry plots showing percentages (gated on CD4+cells) of CD4+FoxP3+T cells in secondary tumours after various treatments indicated. (**c**) Proportions of tumour-infiltrating CD8+ killer T cells, CD4+ FoxP3- effector T cells and CD4+ FoxP3+ regulatory T cells according to data in **a** and **b**. (**d**) CD8+ CTL: Treg ratios and CD4+ effector T cells: Treg ratios in the secondary tumours upon various treatments to remove the first tumours. Both ratios were significantly enhanced after combination treatment with PLGA-ICG-R837-based PTT and anti-CTLA4 therapy. Three mice were measured in each group in **a**–**d**. Data are presented as the mean±s.e.m. Error bars are based on triplicated experiments.

To understand the mechanism of synergistic anti-tumour effect triggered by PLGA-ICG-R837-based PTT in combination with anti-CTLA-4 therapy, immune cells in secondary 4T1 tumours were studied on day 10. While cytotoxic T lymphocytes (CTL) (CD3+CD4-CD8+) could directly kill targeted cancer cells, helper T cells (CD3+CD4+CD8-) play important roles in the regulation of adaptive immunities. In our experiments, for mice with primary tumours removed by surgery, either PLGA-ICG-R837 injection alone or anti-CTLA-4 treatment alone failed to promote CD8+ CTL infiltration into the secondary tumours. In contrast, the percentage of CD8+ CTL in the secondary tumours of mice after the PLGA-ICG-R837-based PTT plus anti-CTLA-4 treatment significantly increased to ∼19.7%, which appeared to be higher than that in groups treated with PLGA-ICG-R837-based PTT (∼8.96%) or surgery plus anti-CTLA4 treatment (∼12.96%) (Fig. 4a,c). On the other hand, compared with the surgery only group, the percentages of helper T cells in the secondary tumours of the other five groups all showed dramatic increase (Fig. 4a,c). Moreover, the percentage of total T cells in the secondary tumour also showed remarkable increase after PLGA-ICG-R837-based PTT (Supplementary Fig. 11).

With Foxp3 as the marker, CD4+ helper T cells could be classified into effective T cells that are helpful to promote immune responses (CD3+CD4+Foxp3-), as well as regulatory T cells (Tregs) (CD3+CD4+Foxp3+) which could hamper effective anti-tumour immune responses. Immune cells in secondary tumours were collected for further analysis after co-staining with CD4 and Foxp3. Among the greatly enriched CD4+ helper T cells in the secondary tumours of mice post PLGA-ICG-R837-based PTT ablation of primary tumours, most of these increased helper T cells were immune-suppressive Tregs. Therefore, although vaccine-like immune responses have been generated after PLGA-ICG-R837-based PTT, the anti-tumour efficacy in this group remained to be less effective owing to the presence of high numbers of Tregs. It was found that CTLA-4 blockade therapy could greatly reduce the percentages of Tregs (CD3+CD4+Foxp3+) in secondary tumours (Fig. 4b). Therefore, both CD8^+^ CTL/Treg ratio and CD4+Teff/Treg ratios were greatly enhanced in secondary tumours of mice after PLGA-ICG-R837-based PTT plus anti-CTLA-4 treatment (Fig. 4c,d). Moreover, comparing the last two groups in Fig. 4 (groups 5 and 6), PLGA-ICG-R837-based PTT plus anti-CTLA4 induced the highest percentage of CD8+ CTLs (also the CD8^+^ CTL/Treg ratio), which are primarily responsible for cell immunity in cancer immunotherapy.

### Long-term immune-memory effects

**Figure 5:**
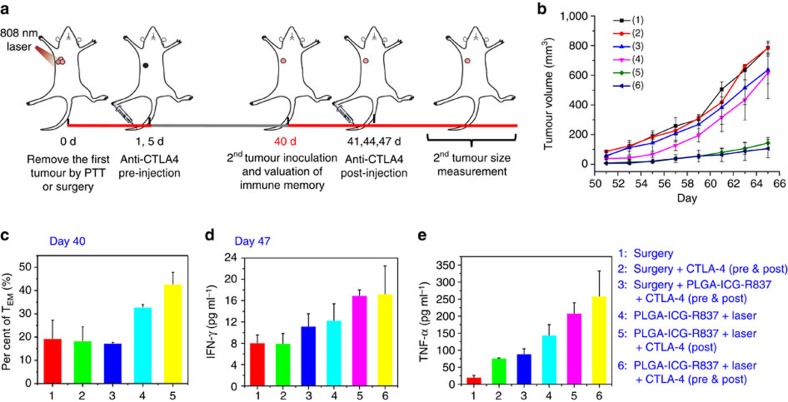
Long-term immune-memory effects. (**a**) Schematic illustration of PLGA-ICG-R837-based PTT and anti-CTLA-4 combination therapy to inhibit cancer relapse. (**b**) tumour growth curves of rechallenged tumours inoculated 40 days post eliminated of their first tumours (eight mice per group). (**c**) Proportions of effector memory T cells (T_EM_) in the spleen analysed by flow cytometry (gated on CD3+ CD8+T cells) at day 40 right before rechallenging mice with secondary tumours (groups 4 and 5 would be identical at this point). (**d**,**e**) Cytokine levels in sera from mice isolated 7 days after mice were rechallenged with secondary tumours (after the second round of anti-CTLA4 treatment). Three mice were measured in each group in **c**–**e**. Data are presented as the mean±s.e.m.

An important feature of immune systems is their ability to remember pathogens for several decades, critical for disease prevention. Therefore, it is necessary to evaluate immune memory induced generated by PLGA-ICG-R837-based PTT. In our experiment, the secondary 4T1 tumours were inoculated 40 days after PLGA-ICG-R837-based PTT or surgery to remove their first 4T1 tumours. Mice were i.v. injected with anti-CTLA-4 antibody at different days (20 μg per mouse each time) for two rounds of treatment, with the first round given right after their first tumours were eliminated (days 1 and 5), and the second round given right after their secondary tumours were re-inoculated (days 41, 44 and 47) (Fig. 5a). It was found that the re-inoculated tumours in mice after their first tumours were ablated by PLGA-ICG-R837-based PTT plus CTLA4 blockade therapy showed inhibited growth (Fig. 5b), and anti-CTLA4 treatment in the first round (after PTT ablation of first tumours) appeared to be not necessary to induce the immune-memory effect. In marked contrast, surgical removal of first tumours plus two rounds of anti-CTLA4 treatment (pre and post), or even anti-CTLA4 (pre and post) in combined with s.c. injected PLGA-ICG-R837, exerted no appreciable inhibitory effect to the rechallenged tumours. Although s.c. injected PLGA-ICG-R837 in combination with anti-CLTA4 therapy could significantly delay the growth of existing tumour cells in the body likely via non-specific immune responses (Fig. 3b,c), such a treatment without generating tumour-associate agents has no immune memory effect to protect mice from tumour rechallenge (Fig. 5b).

We next carried out a series of analyses to understand the robust anti-tumour immune memory generated after PLGA-ICG-R837-based photothermal tumour ablation. Based on the effector function, proliferative capacity and migration potential, memory T cells are classified into central memory T cells (T_CM_) and effector memory T cells (T_EM_) (ref. 57). While T_CM_ mainly locates in the secondary lymphoid tissues and only provides protections after antigen-stimulated clonal expansion, differentiation and trafficking, T_EM_ residing in both lymphoid and non-lymphoid tissues can elicit immediate protections by producing cytokines like IFN-γ (refs 58, 59, 60). Therefore, we measured the proportions of both T_CM_ and T_EM_ cells at day 40 after the removal of the primary tumours with different treatments (right before rechallenging mice with secondary tumours). It was found that the percentage of T_EM_ cells (CD3+CD8+CD62L-CD44+) was much higher in the group of mice with their primary tumour removed by PLGA-ICG-R837-based PTT (Fig. 5c), whereas the percentage of T_CM_ cells (CD3+CD8+CD62L+CD44+) that are less important for immune memory decreased after PLGA-ICG-R837-based PTT together with anti-CTLA treatment in the first round (Supplementary Fig. 12). Furthermore, 1 week after secondary tumours were introduced, cytokines in sera of mice with different treatments were analysed by ELISA. It is known that Th1 cytokines including TNF-α and IFN-γ (ref. 61), the typical markers of cellular immunity, play vital roles in immunotherapy against cancer. The serum levels of TNF-α and IFN-γ were significantly increased in the mice treated with PLGA-ICG-R837-based PTT, particularly for those with PTT plus the second round of anti-CTLA-4 treatment (post), indicating the successful establishment of anti-tumour immune responses triggered by the rechallenging of cancer cells 40 days later in this group (Fig. 5d,e).

### Reformulated nanoparticles for systemic administration

**Figure 6:**
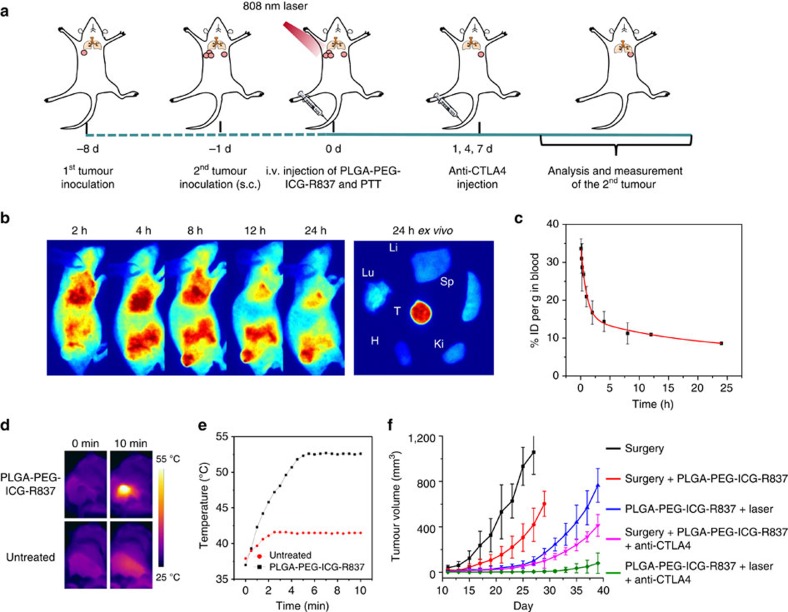
PTT-triggered immunotherapy via systemic injection of nanoparticles. (**a**) Schematic illustration showing the design of animal experiments. (**b**) *In vivo* fluorescence images of CT26-tumour-bearing mice taken at different time points post i.v. injection of PLGA-PEG-ICG-R837. The right column shows an *ex vivo* fluorescence image of major organs and tumour dissected from the mouse 24 h post injection. Tu, Li, Sp, Ki, H and Lu stand for tumour, liver, spleen, kidney, heart and lung, respectively. (**c**) Blood circulation curve of PLGA-PEG-ICG-R837 in mice by measuring the fluorescence of ICG in blood at different time points post i.v. injection (three mice per group). (**d**) IR thermal images of CT26-tumour-bearing mice injected with PLGA-PEG-ICG-R837 or PBS under the 808 nm laser (0.8 W cm^−2^) irradiation. (**e**) The tumour temperature changes based on IR thermal imaging date in **d**. (**f**) The growth curves of secondary tumours in different groups of CT26-tumour-bearing mice after various treatments to eliminate their primary tumours (six mice per group). Data are presented as the mean±s.e.m.

Finally, we would like to further explore the possibility of realizing PTT-triggered cancer immunotherapy by intravenous (i.v.) systemic administration of nanoparticles (Fig. 6a). Polyethylene glycol (PEG) grafted PLGA co-polymer (mPEG-PLGA), instead of PLGA, was used to encapsulate both ICG and R837 (Supplementary Fig. 13). As revealed by *in vivo* and *ex vivo* fluorescence imaging, such PLGA-PEG-ICG-R837 nanoparticles showed rather high accumulation in CT26 tumours upon i.v. injection (Fig. 6b), owing to their prolonged blood circulation behaviour (Fig. 6c) favourable for tumour passive uptake via the enhanced permeability and retention effect.

Utilizing the high tumour accumulation of PLGA-PEG-ICG-R837, photothermal ablation of the first tumour was carried out. After i.v. injection with PLGA-PEG-ICG-R837 (6 mg kg^−1^ R837, 8 mg kg^−1^ ICG) for 24 h, CT26-tumour-bearing mice were irradiated by the 808 nm laser for 10 min (0.8 W cm^−2^), which led to the rise of tumour surface temperature to ∼52 °C (Fig. 6d,e). To evaluate the immunotherapeutic efficacy of PLGA-PEG-ICG-R837-based PTT combined with CTLA-4 blockade, animal experiments were carried out as illustrated in Fig. 6a. After the first tumour of each mouse was removed by PTT (with i.v. injection of PLGA-PEG-ICG-R837) or simply by surgery, the growth of the secondary tumour was carefully monitored (Fig. 6f). As expected, PTT based on PLGA-PEG-ICG-R837 together with CTLA-4 blockade could obviously delay the growth of secondary tumours, offering an obviously prior therapeutic effect compared with other control groups (Fig. 6f).

To further understand the immune effects triggered by PTT with i.v. injected PLGA-PEG-ICG-R837, mice after different treatments were killed and their DCs were collected from the nearest lymph nodes for assessment by flow cytometry (Supplementary Fig. 14). Serum of mice after different treatments was collected at 24, 72 and 168 h post treatment to analyse the changes of various cytokines (Supplementary Fig. 15). It was found that PTT with i.v. injected PLGA-PEG-ICG-R837 was able to induce a high level of DC maturation as well as enhanced secretion of multiple pro-inflammatory key cytokines. Even with systemic administration of our nanoparticles, we did not observe any notable cytokine-storm-like side effect. All mice behaved normally after treatment with i.v. injected PLGA-PEG-ICG-R837 without significant body weight fluctuation or accidental death. Although further studies are still required to carefully evaluate the safety and efficacy of this treatment approach with i.v. injected PLGA-PEG-ICG-R837 nanoparticles, achieving PTT-triggered cancer immunotherapy with systemic injection of all therapeutic agents may have a great value for clinical uses, especially for certain tumours that can hardly be reached via local injection.

## Discussion

Compared with currently existing immunotherapeutic strategies, our method by photothermal tumour ablation with immune-adjuvant nanoparticles together with checkpoint-blockade therapy may overcome several critical issues in cancer immunotherapy. Compared with conventional cancer vaccines with specific proteins or peptides as antigens, whose efficacies may vary significantly between different groups of patients because of the varied antigen expression levels in their tumours, such *in situ* generated ‘tumour vaccines' utilizing tumour residues as tumour-associated antigens after ablation therapy may induce anti-tumour immune responses against a broad spectrum of solid tumours. For traditional whole cancer cell vaccine strategies, cell lysates made from dissected tumour tissues are usually re-injected into patients to generate immune responses. In contrast, our strategy to generate vaccine-like functions *in situ* does not need sophisticated procedures, has no ethic concerns, and is able to efficiently elicit strong protections owing to the synergistic effects between photothermally generated tumour residues and nano-adjuvants pre-injected into the primary tumour, or delivered into tumours after systemic administration. Compared with adoptive T-cell therapy or DC-based therapy, which usually require sophisticated techniques and a lot of hands on experiences, our approach is obvious simpler and cheaper, favourable for future clinical practices. Distinguished from previously reported inorganic materials-based photothermal strategies^62^,^63^, all the components within our nanoparticles have already been approved by US FDA for clinical use. Lastly, although the penetration depth of light in tissues could be limited even with NIR lasers, endoscope-based clinical devices with imbedded laser optical fibres may be used for laser treatment of tumours located deeply inside the body. Therefore the clinical translation of our nanoparticles and proposed technique may indeed be realistic.

In summary, we propose that photothermal ablation of tumours with multifunctional nanoparticles encapsulating both NIR heaters and immune-adjuvant TLR agonists could induce vaccine-like immune responses that may be combined with CTLA4 checkpoint blockade to realize highly effective cancer immunotherapy. Great anti-tumour efficacies have been observed using this strategy to treat two types of subcutaneous tumour models, an artificial whole-body metastasis tumour model, as well as an orthotopic tumour model with spontaneous metastasis. Furthermore, a strong immune-memory effect is observed 40 days later after photothermal tumour ablation with those nanoparticles, which together with anti-CLTA4 therapy would be able to effectively protect mice from tumour rechallenge. Using reformulated nanoparticles, we further demonstrate that such a treatment strategy may also be realized with systemic injection of all agents. Therefore, our study presents a new cancer treatment strategy, which may be able to eliminate primary tumours, attack and kill spreading metastatic tumours, and finally offer immune-memory protection to prevent tumour relapse. Considering that all components in our nanoparticles are FDA-approved ones, our technique may indeed have a good chance for future clinical translation.

## Methods

### Materials

PLGA, ICG, R837(TLR7 ligand) and polyvinyl alcohol (PVA) were obtained from Sigma-Aldrich. Polyethylene glycol (PEG) grafted PLGA co-polymer (mPEG-PLGA), 50:50 (w:w), (Mw ∼5,000:10,000 Da) was purchased from PolySciTech. Dimethyl sulfoxide (DMSO) and dichloromethane (CH_2_Cl_2_) were obtained from Sinopharm Chemical Reagent Co. Anti-CTLA-4 used *in vivo* was obtained from Bioxcell. Antibodies against cell surface markers for flow cytometry (fluorescent-activated cell sorting) assay were purchased from eBioscience.

### Synthesis of PLGA-ICG-R837 nanoparticles

PLGA-ICG-R837 nanoparticles were formed using o/w single-emulsion method^64^. Briefly, R837 (TLR7 ligand) was dissolved in DMSO at 2.5 mg ml^−1^. Photothermal agent ICG was dissolved at 10 mg ml^−1^ in DMSO. A total of 38 μl R837 and 6.25 μl ICG were added to 1 ml PLGA (5 mg ml^−1^) dissolved in dichloromethane. Then, the mixture was homogenized with 0.4 ml 5% w/v PVA solution for 10 min using Selecta Sonopuls. The o/w emulsion was then added to 2.1 ml of a 5% w/v solution of PVA to evaporate the organic solvent for 4 h at room temperature. PLGA-ICG-R837 nanoparticles were obtained after centrifugation at 3,500*g* for 20 min.

### Synthesis of PLGA-PEG-ICG-R837 nanoparticles

PLGA-PEG-ICG-R837 nanoparticles were formulated based on a previously described protocol with slight modifications^65^. Briefly, R837 was dissolved in DMSO at 2.5 mg ml^−1^ and ICG was dissolved in DMSO at 10 mg ml^−1^. 60 μl R837 and 25 μl ICG solutions in DMSO were added to 1 ml mPEG-PLGA (10 mg ml^−1^) dissolved in acetonitrile. Then, the mixture was dropwisely added into 5 ml water. After 1 h stirring and 12 h standing, PLGA-PEG-ICG-R837 nanoparticles were obtained after centrifugation at 22,000*g* for 5 min.

### Nanoparticle characterization

The morphology and structure of PLGA-ICG-R837 were characterized by transmission electron microscopy using a FEI Tecnai F20 transmission electron microscope. The ultraviolet–visible–NIR absorbance spectra were recorded by a PerkinElmer Lambda 750 ultraviolet–visible–NIR spectrophotometer. The dynamic diameters of nanoparticles were determined by a Zetasizer Nano-ZS (Malvern Instruments, UK). The encapsulation efficiency of R837 was determined by a HPLC (Agilent 1260) with a ultraviolet–visible detector at 325 nm. Acetonitrile was used as the mobile phase. The ICG encapsulated in nanoparticles was measured by ultraviolet–visible–NIR.

### Cellular experiments

4T1 murine breast cancer and CT26 colorectal cancer cell lines were originally obtained from American Type Culture Collection (ATCC) and cultured under recommended conditions. Dendritic cells were isolated from the bone marrow of ∼8-week-old BALB/c mice purchased from Nanjing Peng Sheng Biological Technology Co. Ltd. according to an established method^66^. For *in vitro* DC stimulation experiments, DCs were treated with free R837, PLGA-ICG or PLGA-ICG-R837 for 12 h. Alternatively, residues of 4T1 cells after photothermal ablation with either PLGA-ICG or PLGA-ICG-R837 were also added into DC culture using a transwell system. Lipopolysaccharide (LPS, Sigma) at 1 μi ml^−1^ was used as the positive control. After various treatments, DCs were stained with anti-CD11c FITC, anti-CD86 PE and anti-CD80 APC, and then sorted by flow cytometry (BD FACSCalibur).

### *In vivo* experiments

Female BALB/c mice (6–8 weeks) were purchased from Nanjing Peng Sheng Biological Technology Co Ltd and used under protocols approved by Soochow University Laboratory Animal Center. Mice were divided into groups randomly. For the first tumour inoculation, 4T1 cells or CT26 cells (1 × 10^6^) suspended in PBS were subcutaneously injected into the left flank of each female BALB/c mouse. For the second tumour inoculation, which was conducted 7 days later, 4T1 cells (2 × 10^5^) or CT26 cells (4 × 10^5^) suspended in PBS were subcutaneously injected into the right flank of each female BALB/c mouse. The tumor volume was calculated according to the following formula: width^2^ × length × 0.5.

To establish lung metastases, fLuc-4T1 cells (1 × 105), a gift from PerkinElmer Inc., were administered intravenously via tail vein infusion into each BALB/c mouse. Mice were injected with the relevant substrate before bioluminescence imaging, which was carried out using an *in vivo* imaging instruments (IVIS) spectrum system with 60 s exposure time. Besides, lungs were analysed *ex vivo* at different days after i.v. injection of 4T1 tumour cells. Mice were killed right after being injected with India ink through the trachea. Tumour metastasis sites subsequently appeared as white nodules on the surface of black lungs and were counted under a microscope.

To establish 4T1 orthotopic murine breast cancer model with spontaneous metastasis, fLuc-4T1 cells (5 × 10^5^) suspended in PBS were inoculated into the breast pad of each mouse. Two weeks later, the primary tumour on each mouse was removed by PLGA-ICG-R837-based photothermal therapy or surgery. In the following days, mice were imaged by an IVIS spectrum system to monitor the spontaneous metastasis. For anti-metastasis treatment, mice were i.v. injected with 20 μl anti-CTLA-4 in 0.2ml PBS on day 1, 4 and 7, after their primary visible tumours were removed by photothermal therapy or surgery.

To study PTT-triggered cancer immunotherapy upon systemic administration, mice bearing CT26 tumours were i.v. injected with PLGA(-PEG)-ICG-R837 nanoparticles (6 mg kg^−1^ R837, 8 mg kg^−1^ ICG). 24 h later, their first tumours were removed by surgery or PTT (808 nm laser, 0.8 W cm^−2^, 10 min ). For surgery or PTT with nanoparticles i.v. injected with 10 μg anti-CTLA4 in 0.2 mL PBS on days 1, 4 and 7. Afterwards, the growth of the secondary tumour was carefully monitored.

### Cytokine detection

Serum samples were isolated from mice after various treatments and diluted for analysis. Tumour necrosis factor (TNF-α, Dakewe biotech), interferon gamma (IFN-γ, Dakewe biotech), IL-12 (Dakewe biotech) and IL-6 (Dakewe biotech) were analysed with ELISA kits according to vendors' protocols.

### *Ex vivo* analysis of different groups of T cells

To study the immune cells in secondary tumours, tumours were harvested from mice in different groups and stained with anti-CD3-FITC (Biolegend, Clone: 17A2, Catalog: 100204), anti-CD8a-APC (Biolegend, Clone: 53-6.7, Catalog: 100712), anti-CD4-PerCP (Biolegend, Clone: GK1.5, Catalog: 100432) antibodies according to the manufacturer's protocols. Briefly, tumour tissues were cut into small pieces and put into a glass homogenizer containing PBS (pH7.4) with 2% heat-inactivated fetal bovine serum^67^. Then, the single-cell suspension was prepared by gentle pressure with the homogenizer without addition of digestive enzyme. Finally, cells were stained with fluorescence-labelled antibodies after the removal of red blood cells (RBC) using the RBC lysis buffer. Cytotoxic T lymphocytes (CTL) and helper T cells were CD3+CD4-CD8+ and CD3+CD4+CD8-, respectively. To analyse CD4+ helper T cells, cells in the secondary tumour were further stained with anti-CD3-FITC (eBioscience, Clone: 145-2C11, Catalog: 11-0031), anti-CD4-PerCP (Biolegend, Clone: GK1.5, Catalog: 100432), and anti-Foxp3-PE (eBioscience, Clone: NRRF-30, Catalog: 12-4771) antibodies according to the standard protocols. CD4+ helper T cells were classified into effective T cells (CD3+CD4+Foxp3-) and regulatory T cells (Tregs) (CD3+CD4+Foxp3+). For analysis of memory T cells, lymph nodes harvested from mice after various treatment were stained with anti-CD3-FITC (eBioscience, Clone: 145-2C11, Catalog: 11-0031), anti-CD8-PerCP-Cy5.5 (eBioscience, Clone: 53-6.7, Catalog: 45-0081), anti-CD62L-APC (eBioscience, Clone: MEL-14, Catalog: 17-0621) and anti-CD44-PE (eBioscience, Clone: IM7, Catalog: 12-0441) antibodies according to the manufacturer) antibodies. The single-cell suspension from lymph nodes was prepared using the same protocol to that of tumour tissues^67^. Central memory T cells (T_CM_) and effector memory T cells (T_EM_) were CD3+CD8+CD62L+CD44+ and CD3+CD8+CD62L-CD44+, respectively. All these antibodies used in our experiments were diluted ∼200 times.

### Data availability

All relevant data are available from the authors.

## Additional information

**How to cite this article:** Chen, Q. *et al*. Photothermal therapy with immune-adjuvant nanoparticles together with checkpoint blockade for effective cancer immunotherapy. *Nat. Commun.* **7,** 13193 doi: 10.1038/ncomms13193 (2016).

## Supplementary Material

Supplementary Information Supplementary Figures 1-15 and Supplementary Table 1.
