# Supplementary material for: Photothermal therapy with immune-adjuvant nanoparticles together with checkpoint blockade for effective cancer immunotherapy
Source: Nat Commun. 2016 Oct 21;7:13193. doi: 10.1038/ncomms13193 (PMC5078754; doi:10.1038/ncomms13193)
Supplement: Supplementary Information — Supplementary Figures 1-15 and Supplementary Table 1. [file ncomms13193-s1.pdf]

Supplementary Information

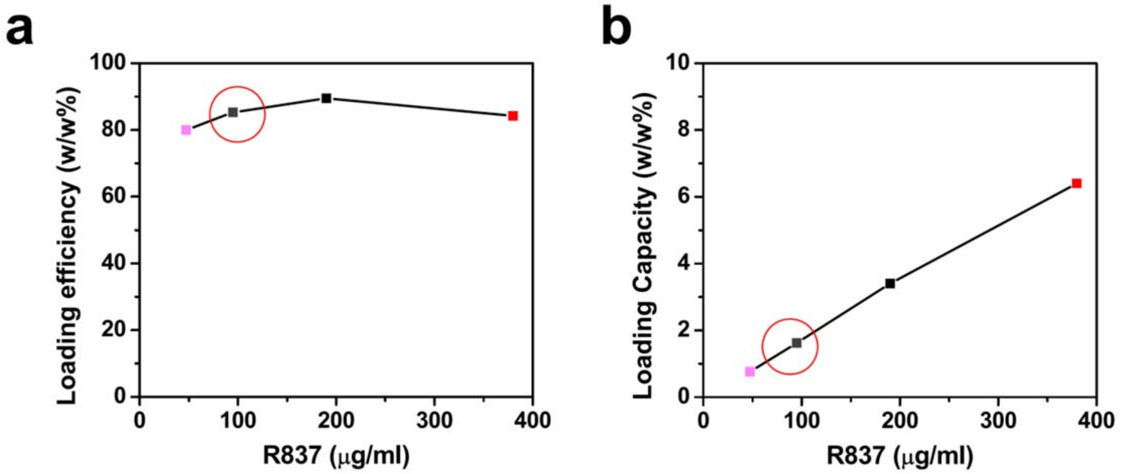

Supplementary Figure 1. Loading of R837 into PLGA nanoparticles. The loading efficiency (a) and loading capacity (b) of R837 by PLGA nanoparticles obtained at different feeding concentrations measured by HPLC. The concentration of R837 was fixed at 100 μg/mL in this experiment

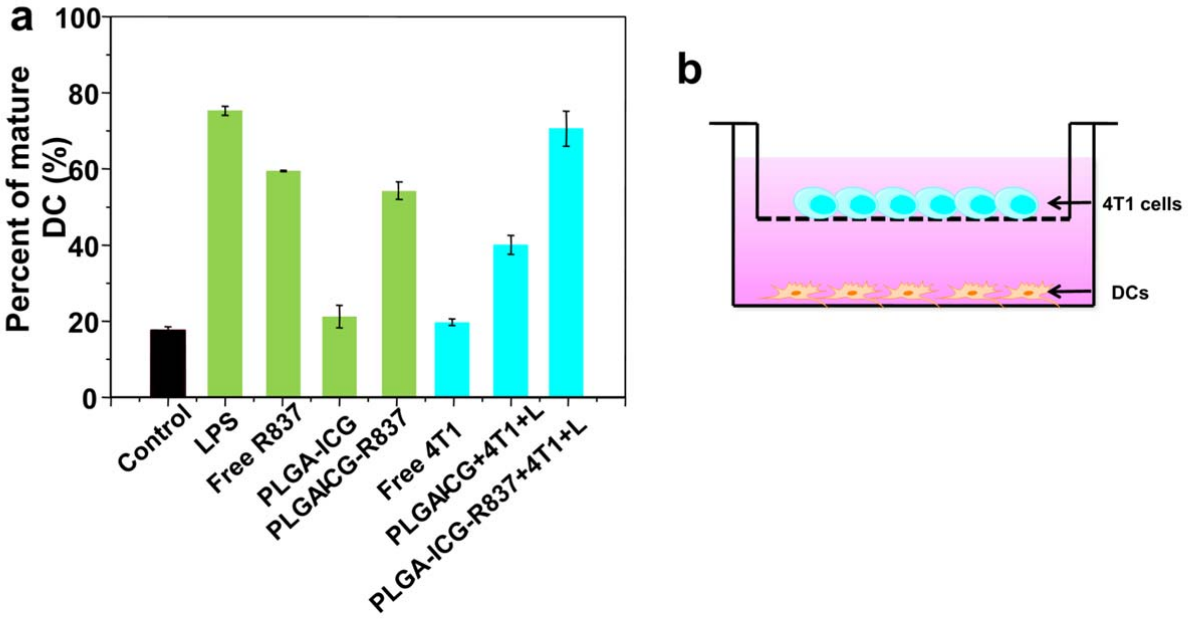

Supplementary Figure 2. Maturation of bone marrow-derived DCs. **(a)** The percent of mature DCs (CD11c+CD80+CD86+) separated from Balb/c mice after incubation with lipopolysaccharide (LPS), free R837, PLGA-ICG and PLGA-ICG-R837 for 12 h. In another set of experiments, DCs were co-cultured with 4T1 breast tumor cells, or with residues of 4T1 cells after NIR laser induced photothermal ablation with PLGA-ICG or PLGA-ICG-R837 nanoparticles, for 12 h using transwell co-culture systems. DCs were stained with antibodies to label CD11c, CD80, and CD86, and then analyzed by flow cytometry. (b) A scheme illustrating the transwell co-culture system. Error bars were based on standard deviations (SD) of three parallel samples.

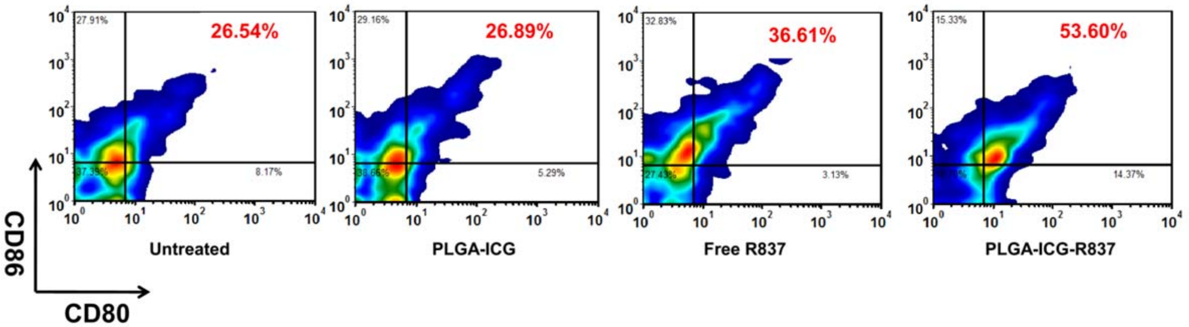

Supplementary Figure 3. In vivo DC maturation induced by PLGA-ICG-R837. BALB/c mice were s.c. injected with PLGA-ICG, Free R837 or PLGA-ICG-R837. Cells in the nearest lymph nodes were examined for the expression of CD11c, CD80, and CD86 by flow cytometry.

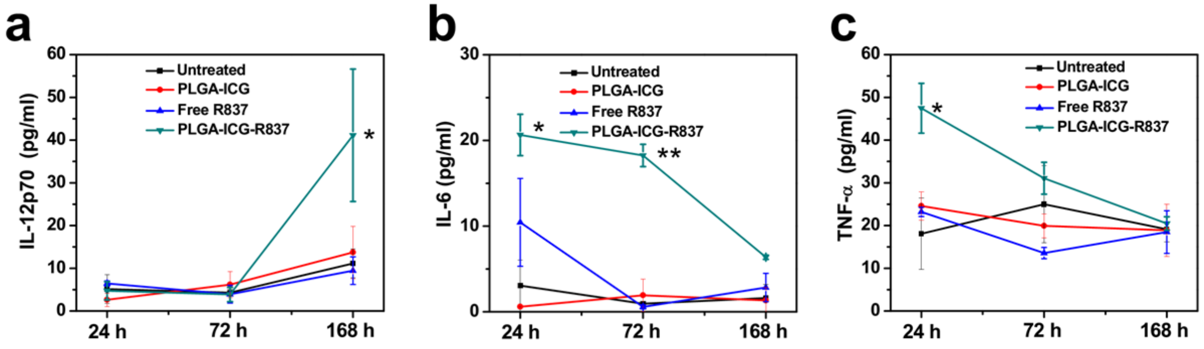

Supplementary Figure 4. Cytokine levels in sera from mice after different treatments. PLGA-ICG-R837 injection enhanced the secretion of IL12p70 (a), IL6 (b), and TNF-α (c). Error bars were based on standard errors of the mean (SEM) of three mice per group. P values were determined between PLGA-ICG-R837 treated group and untreated group.

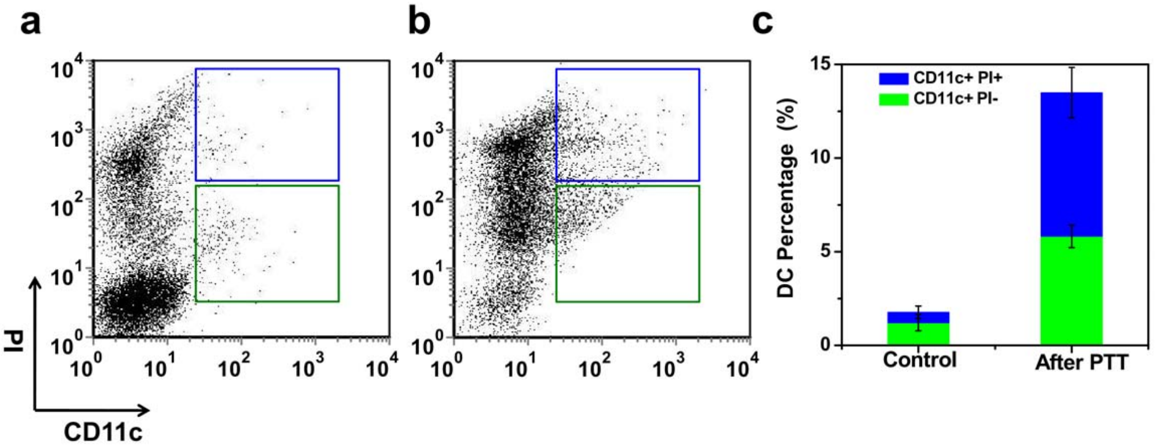

Supplementary Figure 5. The status of DCs in treated tumors after PTT. BALB/c mice bearing 4T1 tumors 4 h after PTT were scarified and their tumor cells were collected and stained with CD11c and PI for assessment by flow cytometry. It seems that after photothermal ablation of the tumor with PLGA-ICG-R837 nanoparticles, more DCs would be recruited into the first tumor site, although DCs pre-existing in the tumor before treatment should have been killed alongside the tumor. Error bars are based on three mice per group.

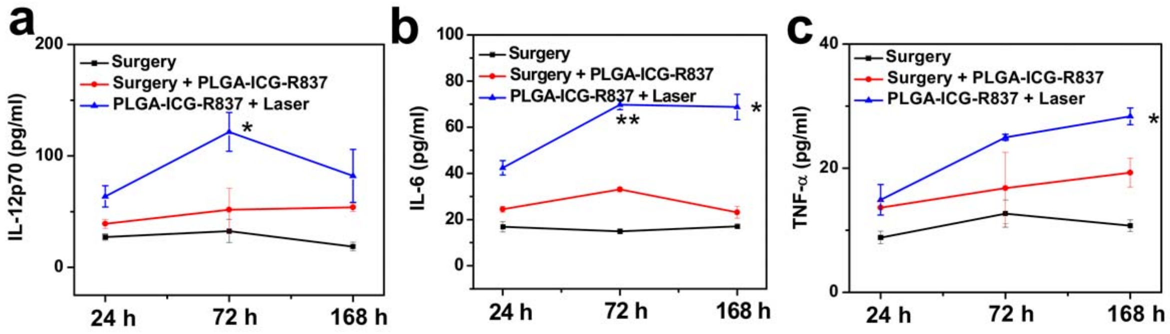

Supplementary Figure 6. The expression of pro-inflammatory cytokines on CT26 tumor model. Cytokine levels including IL12p70 (a), IL6 (b), and TNF-α (c) in sera from mice isolated at 24 h, 72 h and 168 h post different treatments. Although the kinetics of cytokine secretion could vary between mice bearing different tumor models (Figure 2 i-k) as well as healthy mice (Figure S4), the general trends appeared to be consistent. P values were determined between the second group (Surgery + PLGA-ICG-R837) and the third group (PLGA-ICG-R837 + Laser). Error bars are based on three mice per group.

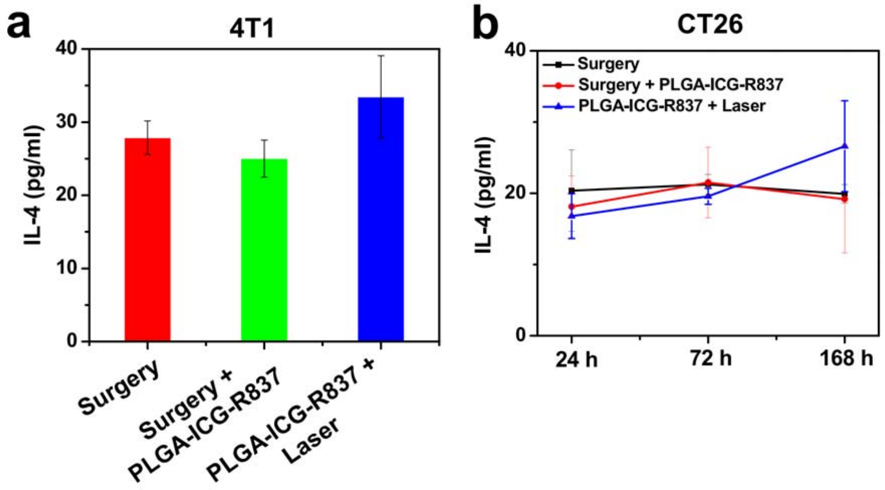

Supplementary Figure 7. IL4 levels in sera of mice with different tumor models. **(a)** IL4 levels in sera from BALB/c mice bearing 4T1 tumors isolated at 72 h after different treatment. **(a)** IL4 levels in sera from BALB/c mice bearing CT26 tumors isolated at 24 h, 72 h and 168 h after different treatment. Error bars are based on three mice per group.

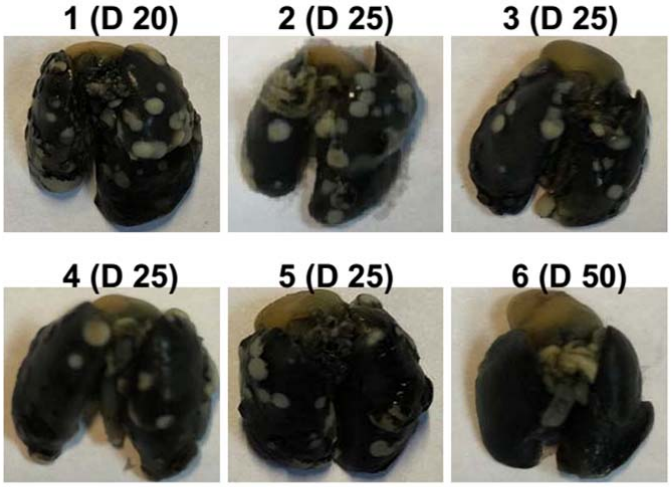

Supplementary Figure 8. Representative lung photographs collected from mice at different time points post various treatments in Figure 3d. White nodules were metastatic tumors in lungs.

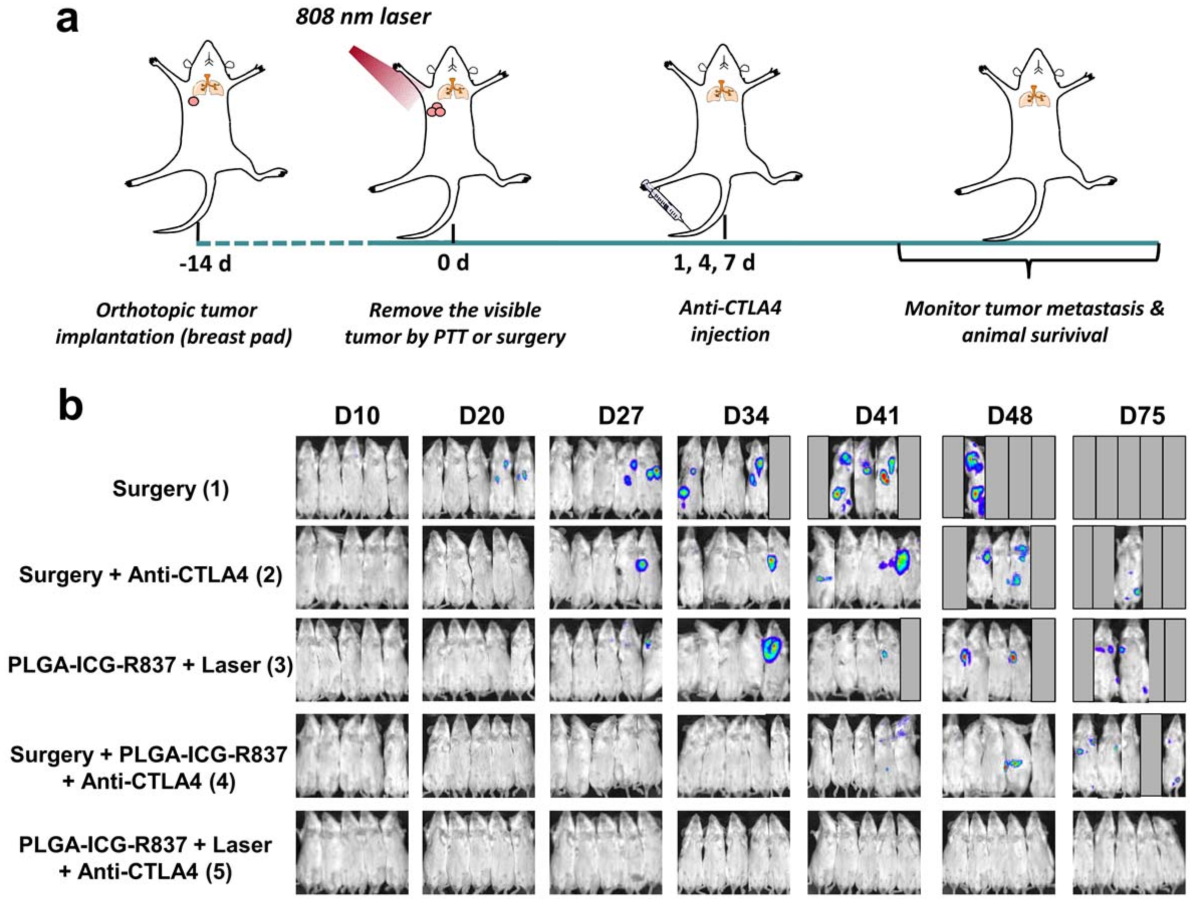

Supplementary Figure 9. Anti-cancer metastasis treatment on the orthotopic breast tumor model. (a) Schematic illustration of PLGA-ICG-R837-based PTT and anti‒CTLA-4 combination therapy to inhibit spontaneous tumor metastases in the orthotopic 4T1 tumor model. (b) In vivo bioluminescence images to track the cancer metastasis in different groups of mice after various treatments to eliminate their primary orthotopic tumors.

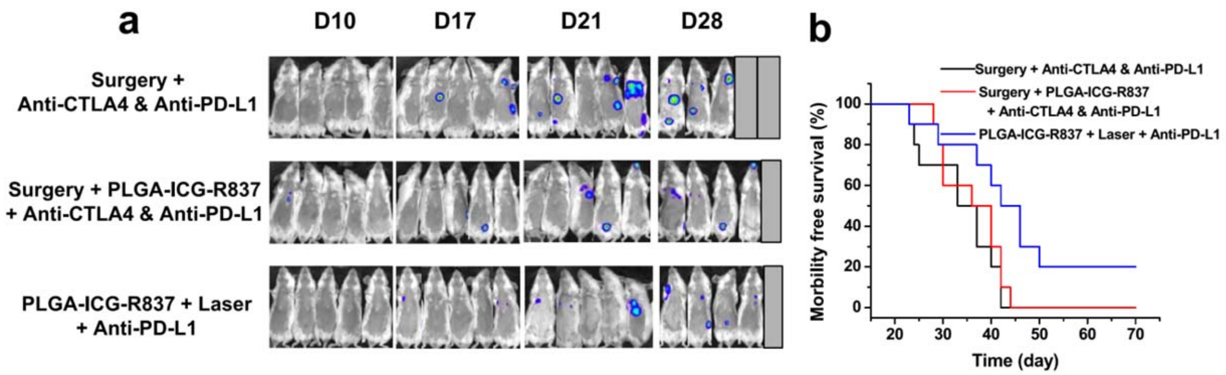

Supplementary Figure 10. Checkpoint blockade with anti-PD-L1. (a) In vivo bioluminescence images to track the spreading and growth of i.v. injected fLuc-4T1 cancer cells in different groups, including surgical removal of subcutaneous tumors plus anti-CTLA-4 + anti-PD-L1, surgery plus anti-CTLA-4 & anti-PD-L1 plus PLGA-ICG-R837, and PLGA-ICG-R837 + Laser (to ablate subcutaneous tumors) plus anti-PD-L1. Spreading of tumor cells appeared in all of those groups. Note that photothermal ablation of subcutaneous tumors with PLGA-ICG-R837 plus co-blockage of CTLA-4 and PD-L1 resulted in death of more than a half of treated mice, likely due to the triggered too violent immune responses. (b) Morbidity free survival of mice after various treatments indicated to eliminate their subcutaneous tumors (10 mice per group).

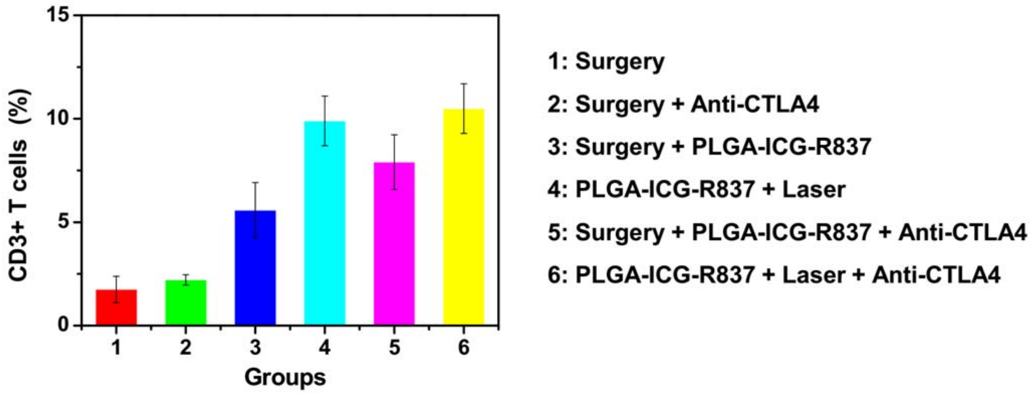

Supplementary Figure 11. Proportions of T cells (CD3+) in the secondary tumors at day 10. Significantly more T cells were infiltrated into the secondary tumor for those with injection of PLGA-ICG-R837 nanoparticles. Error bars are based on three mice per group.

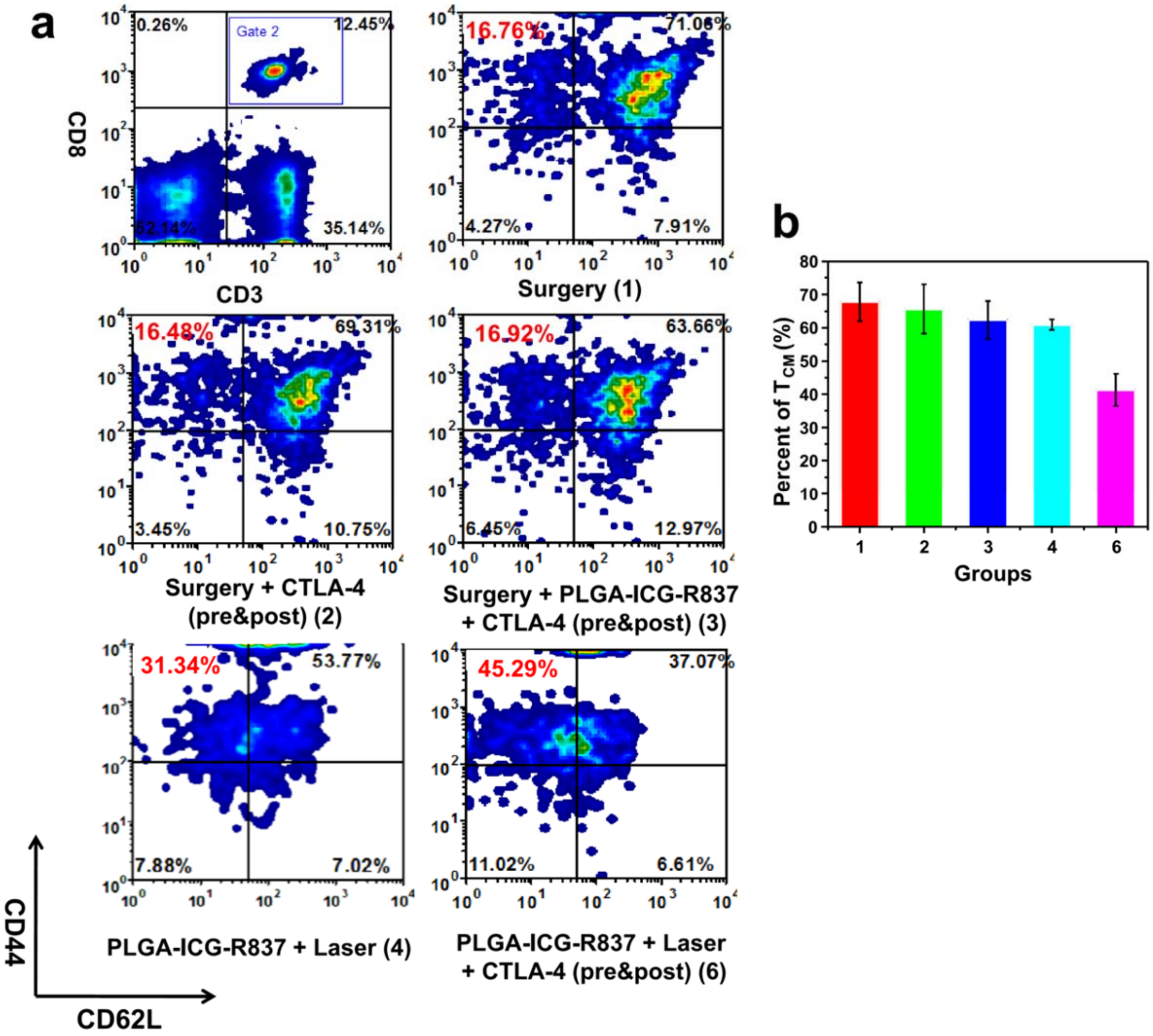

Supplementary Figure 12. Long-term immune-memory effects. (a) The proportions of TCM or TEM cells at day 40 after the removal of the primary tumors with different treatments in the spleen of mice analyzed by flow cytometry for T cell infiltration (gated on CD3+CD8+ T cells). (b) Proportion of central memory T cells (T_{CM}) in the spleen according to (a). Error bars are based on three mice per group.

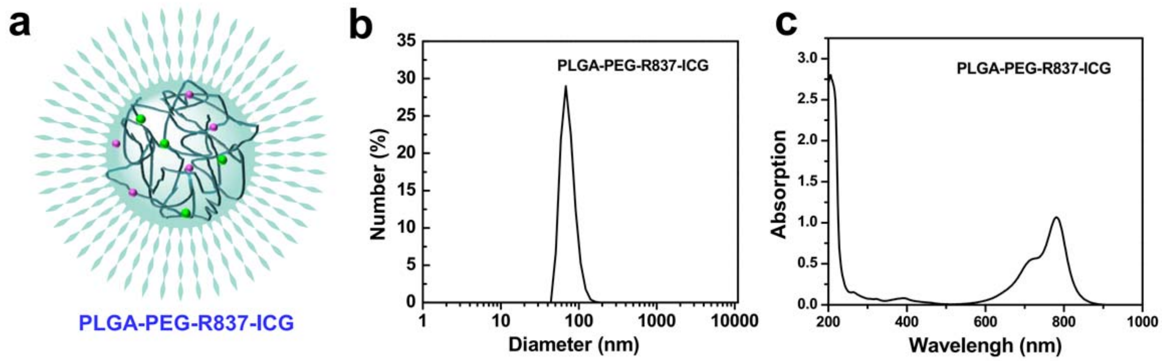

Supplementary Figure 13. Characterization of PLGA-PEG-ICG-R837. (a) Scheme of PLGA-PEG-ICG-R837. (b&c) Hydrodynamic diameters (b) and UV-vis-NIR spectra (c) of PLGA-PEG-ICG-R837 nanoparticles.

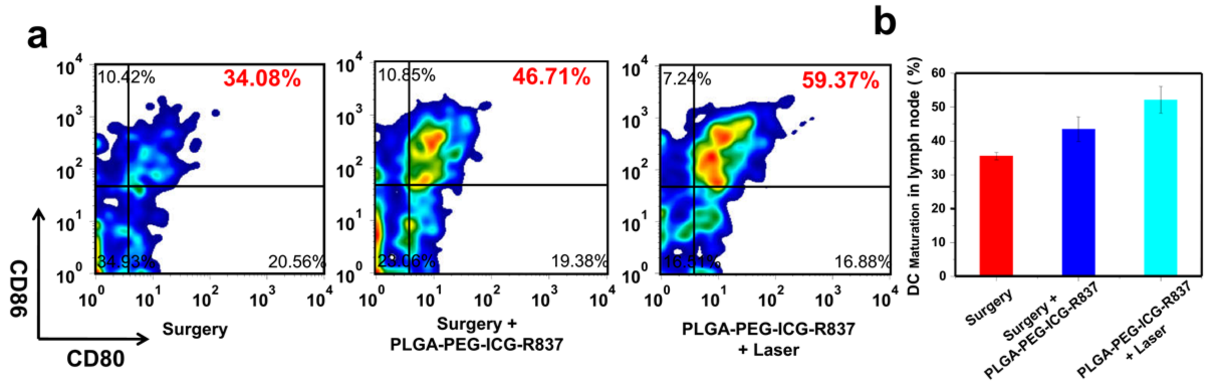

Supplementary Figure 14. DC maturation induced by PTT after i.v. injection with PLGA-PEG-ICG-R837. Cells in the tumor-draining lymph nodes were collected for assessment 72 h post various treatment by flow cytometry after staining with CD11c, CD80, and CD86. Error bars are based on three mice per group.

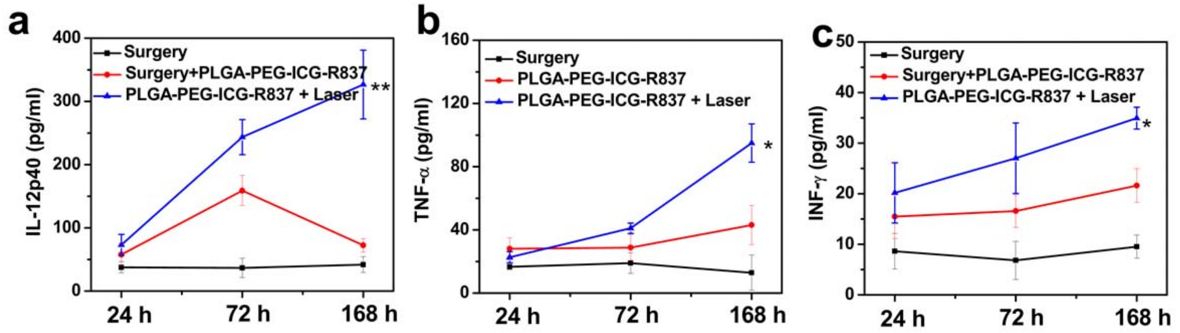

Supplementary Figure 15. The cytokines in sera induced by PTT after i.v. injection of PLGA-PEG-ICG-R837. Cytokine levels including IL12p40 (a), TNF-α (b), and INF-γ (c) in sera from mice isolated at 24 h, 72 h and 168 h after different treatments. P values were determined between the second group (Surgery + PLGA-PEG-ICG-R837) and the third group (PLGA-PEG-ICG-R837 + Laser). Error bars are based on three mice per group.

		WBC (10^9/L)	RBC (10^{12}/L)	HGB（g/L）	HCT（%）	MCV (fL)	MCH (pg)
Reference range		5.69~14.84	8.16~11.69	124~189	43~67	50.8~64.1	13~17.6
Healthy control		9.27±1.54	10.80±0.32	167.25±10.11	46.76±0.81	53.33±0.91	15.50±0.36
1D	Surgery	8.18±1.87	10.04±0.55	153.34±9.54	45.31±0.99	53.13±0.40	15.23±0.12
	PTT	12.9±1.44	10.15±0.66	150.25±8.17	44.16±0.45	53.56±2.07	14.76±0.40
7D	Surgery	11.3±10.84	11.17±0.17	159.33±17.01	48.63±2.20	52.57±1.32	14.96±0.55
	PTT	8.3±1.4	10.37±0.38	145.33±7.76	46.37±1.80	54.63±0.71	15.33±0.21
14D	Surgery	12.3±0.56	10.63±0.11	161.67±0.57	45.70±0.65	52.96±0.35	15.20±0.10
	PTT	10.39±1.55	10.19±0.32	153.67±5.51	44.46±0.45	53.57±0.98	15.07±0.57

		MCHC (g/L)	PLT (10^9/L)	BUN (mmol/L)	ALT (U/L)	ALP (U/L)	AST (U/L)
Reference range		239~331	476~1611	7~31	40~170	108~367	67~381
Healthy control		257.33±5.03	1005.33±44.65	15.30±0.65	71.93±4.37	148.67±29.85	129.83±19.01
1D	Surgery	253.34±6.35	867.00±81.55	14.13±0.60	63.56±2.05	204.45±16.30	159.33±11.07
	PTT	259.45±10.96	792.00±92.97	14.88±0.56	64.96±13.26	147.62±3.57	236.33±35.23
7D	Surgery	263.67±11.97	1301.00±3.64	15.90±2.67	67.27±3.98	160.50±8.18	212.50±54.83
	PTT	243.78±2.03	1418.00±150.53	15.01±0.32	59.67±11.99	190.58±19.13	140.50±14.00
14D	Surgery	254.23±5.29	1120.30±89.97	14.50±0.10	69.33±3.23	196.33±19.79	115.00±10.04
	PTT	246.89±2.72	1011.33±68.37	14.95±0.89	68.27±8.84	163.47±10.56	152.46±21.45

Supplementary Table 1. Complete blood panel and serum biochemistry data. Balb/c mice bearing 4T1 tumor sacrificed at 1, 7, 14 days after surgery or PTT (with PGLA-ICG-R837). Untreated healthy mice were used as the control. Complete blood counts: Blood levels of White blood cells (WBC), Red blood cells (RBC), Hemoglobin (HGB), Hematocrit (HCT), Mean corpuscular volume (MCV), Mean corpuscular hemoglobin (MCH), Mean corpuscular hemoglobin concentration (MCHC). Serum biochemistry data including blood urea nitrogen (BUN) levels and liver function markers such as Alanine aminotransferase (ALT), Alkaline phosphatase (ALP), and Aspartate aminotransferase (AST), were also measured. Reference ranges of hematology data of healthy female Balb/c mice were obtained from Charles River Laboratories :(http://www.criver.com/). Five mice were used in each group for this experiment.
